# Superiority of SpiroZin2 Versus FluoZin-3 for monitoring vesicular Zn^2+^ allows tracking of lysosomal Zn^2+^ pools

**DOI:** 10.1038/s41598-018-33102-w

**Published:** 2018-10-09

**Authors:** Yu Han, Jacob M. Goldberg, Stephen J. Lippard, Amy E. Palmer

**Affiliations:** 1 Department of Biochemistry and BioFrontiers Institute, University of Colorado, Boulder, CO 80303 USA; 2 Department of Chemistry, Massachusetts Institute of Technology, Cambridge, MA 02139-4307 USA

## Abstract

Small-molecule fluorescent probes are powerful and ubiquitous tools for measuring the concentration and distribution of analytes in living cells. However, accurate characterization of these analytes requires rigorous evaluation of cell-to-cell heterogeneity in fluorescence intensities and intracellular distribution of probes. In this study, we perform a parallel and systematic comparison of two small-molecule fluorescent vesicular Zn^2+^ probes, FluoZin-3 AM and SpiroZin2, to evaluate each probe for measurement of vesicular Zn^2+^ pools. Our results reveal that SpiroZin2 is a specific lysosomal vesicular Zn^2+^ probe and affords uniform measurement of resting Zn^2+^ levels at the single cell level with proper calibration. In contrast, FluoZin-3 AM produces highly variable fluorescence intensities and non-specifically localizes in the cytosol and multiple vesicular compartments. We further applied SpiroZin2 to lactating mouse mammary epithelial cells and detected a transient increase of lysosomal free Zn^2+^ at 24-hour after lactation hormone treatment, which implies that lysosomes play a role in the regulation of Zn^2+^ homeostasis during lactation. This study demonstrates the need for critical characterization of small-molecule fluorescent probes to define the concentration and localization of analytes in different cell populations, and reveals SpiroZin2 to be capable of reporting diverse perturbations to lysosomal Zn^2+^.

## Introduction

Zinc is the second most abundant transition metal in mammals and an essential nutrient required for growth. Most intracellular Zn^2+^, concentrations of which are typically hundreds of micromolar in mammalian cells^1^, is tightly bound to proteins. As much as 10% of the human proteome has been predicted to bind Zn^2+^ ions^2^. In these Zn^2+^-containing proteins, the ion serves as a structural component, stabilizing the three-dimensional fold or serving as a catalytic cofactor^1^. The remaining intracellular Zn^2+^ is loosely bound to small-molecule, peptide, and protein ligands and accumulates in pools that are readily exchangeable to maintain Zn^2+^ homeostasis^3^. Additionally, Zn^2+^ may be released from labile pools as a signaling agent^4^, although the mechanisms of Zn^2+^ utilization in sensing are less well understood.

Labile Zn^2+^ pools occur in the cytosol, discrete organelles, and within vesicles of secretory cells^5^, and diverse patterns of dynamics have been observed for these pools. In some regions of the brain, for example, presynaptic glutamatergic vesicles co-release glutamate and Zn^2+^ into the synaptic cleft during neurotransmission, where it modulates the excitatory post-synaptic current by binding to ion channels ostensibly as part of a gain control mechanism^6,7^. Mitochondria in primary rat hippocampal neurons can transiently accumulate Zn^2+^ upon treatment with glutamate and Zn^2+^, suggesting that mitochondria may serve as a temporary store of labile Zn^2+^ ^8^. Zn^2+^ accumulation in lysosomes has been suggested to play roles in oxidative neuronal death and progressive cell degeneration in neurodevelopmental diseases^9,10^. During fertilization, mammalian egg cells release “Zn^2+^ sparks” from intracellular vesicular stores that appear to play crucial roles in ovum activation^11^. Furthermore, in breast cancer cells, Zn^2+^ mobilized from intracellular stores increases the phosphorylation of tyrosine kinases^12^, implicating these pools in a distinct form of Zn^2+^-dependent cell signaling. Finally, mouse mammary epithelial cells form Zn^2+^-rich vesicles in response to lactation hormone treatment^13^, although the mechanism(s) regulating these changes and the identity of the vesicular pools are not well understood. In order to understand the roles of labile Zn^2+^ and the factors that control its homeostasis in these and other cellular events, it is necessary to be able to record the dynamics and distribution of Zn^2+^ in subcellular compartments with high accuracy and precision.

Current tools to monitor labile Zn^2+^ include fluorescent protein (FP)-based sensors and small-molecule chemical probes. FP-based sensors are genetically encodable, and can be specifically targeted to organelles by incorporation of a signal sequence. They have been used to estimate the concentration of labile Zn^2+^ in the ER, Golgi, mitochondria, and nucleus^14–19^. However, measuring Zn^2+^ in vesicular compartments with FP-based probes has been more challenging as the currently available protein-based sensors suffer from low dynamic range in vesicles in response to Zn^2+^ perturbation^17,18^. A growing number of fluorescent small molecule probes have been developed to measure vesicular Zn^2+^ pools, including Zinquin^20^, FluoZin-3^21^, ZincBY-1^11^, SpiroZin1^22^, and SpiroZin2^23^. Many of these probes exhibit large dynamic ranges and they employ diverse mechanisms for detecting Zn^2+^ ions.

In this study, we performed a systematic evaluation of two small-molecule probes, FluoZin-3 AM and SpiroZin2, with an emphasis on comparing the variability of the fluorescence intensities and subcellular distributions of the two dyes in response to identical Zn^2+^ perturbations. FluoZin-3 AM has been widely used to measure vesicular Zn^2+^ in many different mammalian cells^9,10,13,24^. Despite this broad application, FluoZin-3 AM has been reported to exhibit variable intracellular localization in both the cytosol and vesicles, as well as large variability in fluorescence intensity^25^. SpiroZin2 is a red-shifted probe that is insensitive to changes in pH between pH 3 and 7 and has been used to image lysosomal Zn^2+^ in HeLa cells and acute hippocampal tissue slices^23^. We find here that FluoZin-3 AM exhibits highly variable fluorescence intensities and non-specifically localizes in the cytosol and in multiple vesicular compartments. By comparison, SpiroZin2 produced consistent and uniform fluorescence intensities in different cells. In addition, we find that SpiroZin2 specifically localizes in late endosome/lysosome vesicles, making it an ideal probe to estimate lysosomal labile Zn^2+^ levels. We then used SpiroZin2 to detect changes in lysosomal Zn^2+^ pools upon manipulation of extracellular Zn^2+^, dissipation of the lysosomal pH gradient, activation of the lysosomal channel TRPML1, and differentiation of mouse mammary epithelial cells (HC11).

## Results

**Figure 1:**
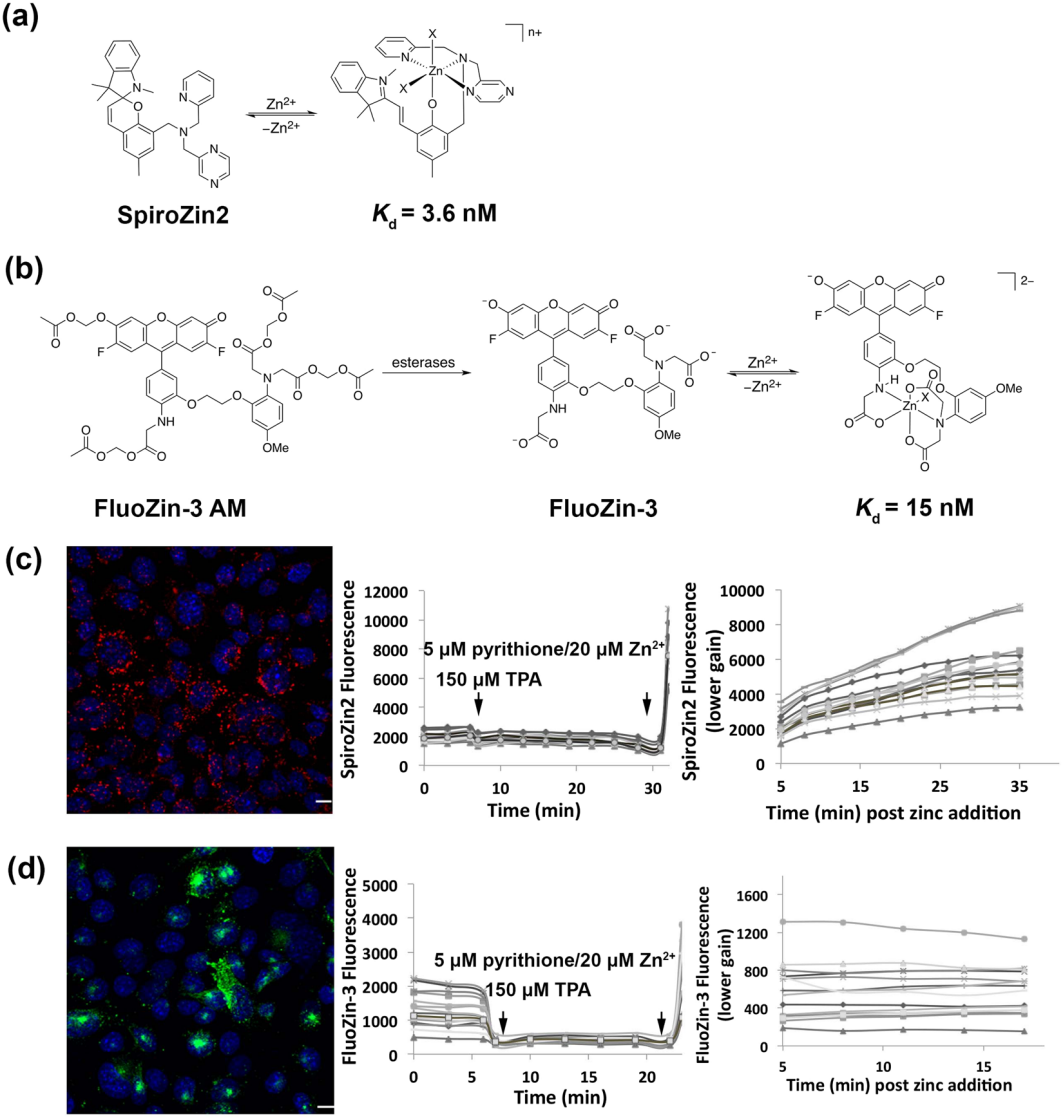
Titration of probes in HC11 cells. (**a**) Molecular structure of zinc-unbound and zinc-bound SpiroZin2 λ ex = 520 nm, λ em = 640 nm. (**b**) Molecular structure of FluoZin-3 AM, zinc-unbound and zinc-bound FluoZin-3, λ ex = 494 nm, λ em = 516 nm. (**c**) Representative image of SpiroZin2-stained HC11 cells and the titration curve of SpiroZin2 in HC11 cells. Red: SpiroZin2. Blue: nucleus. To avoid saturation of pixels, lower gain was applied to acquire images post zinc addition. (**d**) Representative image of FluoZin-3-stained HC11 cells and the titration curve of FluoZin-3 in HC11 cells. Green: FluoZin-3. Blue: nucleus. To avoid saturation of pixels, lower gain was applied to acquire images post zinc addition. Scare bar, 10 μm.

Because FluoZin-3 and SpiroZin2 employ different fluorescence sensing mechanisms, we performed a side-by-side comparison of their response to Zn^2+^ perturbations in HC11 cells. Figure 1 depicts the chemical structures of FluoZin-3 and SpiroZin2, along with representative fluorescence images and Zn^2+^ response curves. In these experiments, we used the cell-permeable acetoxymethyl (AM) ester derivative of FluoZin-3, FluoZin-3 AM, in which the negatively charged carboxylate groups are masked as neutral esters. After the functionalized dye crosses the cell membrane, intracellular esterases hydrolyze the esters, restoring the carboxylic acids and trapping the negatively charged probe inside the cell. In the absence of Zn^2+^, FluoZin-3 fluorescence is quenched by photoinduced electron transfer (PET) from the chelating group. Upon Zn^2+^ binding, PET becomes unfavorable, which causes an increase in the fluorescence emission^21^. In contrast, SpiroZin2 operates via a reaction-based turn-on mechanism that occurs when Zn^2+^ binding induces a ring-opening reaction that converts a non-fluorescent spirobenzopyran to a fluorescent cyanine dye^23^. Figure 1(c,d) show representative fluorescence images of HC11 mouse mammary epithelial cells stained with SpiroZin2 and FluoZin-3, respectively. Cells treated with SpiroZin2 routinely exhibited fluorescence in a vesicular pattern. FluoZin-3 fluorescence signals were more variable, appearing to reside in vesicles, large puncta, and the cytosol.

In order to measure the response of each probe to Zn^2+^ perturbation, cells stained with either SpiroZin2 or FluoZin-3 were treated with reagents to determine the minimum and maximum fluorescence signal of the apo and Zn^2+^-bound states. To obtain the minimum fluorescence signal for the apo form of the sensor (F_min_), the cell-permeable Zn^2+^ chelator tris(2-pyridylmethyl)amine (TPA) was added to cells. Subsequently, the maximal fluorescence signal for the bound form of the sensor (F_max_) was obtained by addition of excess Zn^2+^ with the ionophore pyrithione. The titration curves in Figure 1(c,d) demonstrate that both SpiroZin2 and FluoZin-3 respond to Zn^2+^ chelating and saturating reagents within a few minutes in HC11 cells.

Single cell analysis of live cells treated with either FluoZin-3 or SpiroZin2 revealed significant heterogeneity in the fluorescence intensities and staining patterns of each dye. Although the fluorescence intensity of FluoZin-3 has been used widely to estimate intracellular resting labile Zn^2+^ levels under resting conditions^26–30^, evidence suggests that the high fluorescence signal of FluoZin-3 in HeLa cells is at least partially due to accumulation of the dye in the Golgi complex rather than high concentrations of labile Zn^2+^ ^25^. Moreover, variability in the amount of dye internalized by cells causes inconsistencies in resting fluorescence signals (F), and, therefore, estimated labile Zn^2+^ levels. This behavior is a notorious issue with all intensity-based dyes: the fluorescence signal (F) depends not only on the analyte of interest (in this case Zn^2+^), but also on the amount of dye present in a cell or in an organelle. We proposed that labile Zn^2+^ levels could be assessed more precisely by normalizing resting state fluorescence to apo state fluorescence after treatment with TPA (F/F_min_), where F_min_ provides a Zn^2+^-independent indicator of the amount of dye in each cell. In this case, greater F/F_min_ ratios would indicate higher concentrations of labile Zn^2+^.

**Figure 2:**
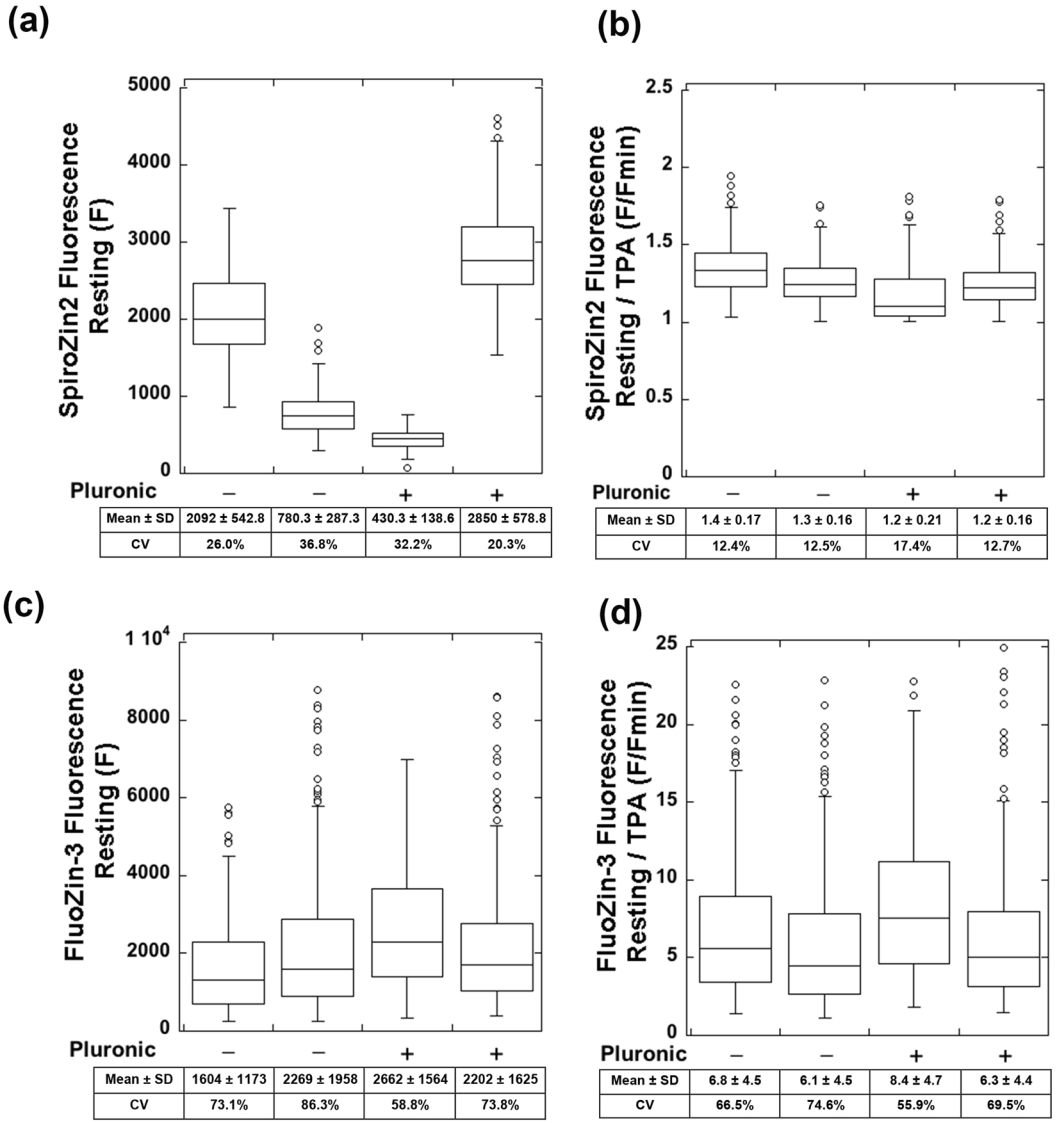
SpiroZin2 staining yields consistent signals after normalization to TPA treatment whereas FluoZin3-stained cells have highly variable signals. (**a**) SpiroZin2 fluorescence at resting state with and without the addition of pluronic. (**b**) SpiroZin2 signal at resting state normalized to TPA treatment with and without the addition of pluronic. (**c**) FluoZin-3 fluorescence at resting state with and without the addition of pluronic. (**d**) FluoZin-3 signal at resting state normalized to TPA treatment with and without the addition of pluronic. Normalization to TPA treatment was accomplished by dividing the respective probe fluorescent intensity by the fluorescence intensity upon TPA treatment for each cell. Each point is an individual cell, n > 62 for each sample. Two biological replicates per condition. SD, standard deviation. CV, coefficient of variation.

To quantify the cellular heterogeneity of fluorescence signals of the two probes, we measured the fluorescence intensities of single HC11 cells stained with either SpiroZin2 or FluoZin-3 AM, with or without pluronic F-127, before (F) and after treatment with TPA (F_min_). Pluronic F-127 is a non-ionic detergent that facilitates the dispersion of hydrophobic dyes in aqueous media and is often applied in standard FluoZin-3 staining protocols. Figure 2(a,c) shows that both SpiroZin2 and FluoZin-3 staining resulted in highly variable resting fluorescence (F). In contrast, the normalized fluorescence (F/F_min_) of SpiroZin2 gave consistent results across multiple trials. For example, in two independent experiments in the absence of pluronic, we found F/F_min_ = 1.4 ± 0.17 and 1.3 ± 0.16. In two separate experiments, we found that co-incubation with pluronic F-127 gave similar results: F/F_min_ = 1.2 ± 0.21 and 1.2 ± 0.16. These data suggest that the ratio F/F_min_ is a better indicator of resting Zn^2+^ levels than F. FluoZin-3 staining experiments gave a large range of F/F_min_ (ratios = 6.8 ± 4.5 and 6.1 ± 4.5 for two independent trials in the absence of pluronic; 8.4 ± 4.7 and 6.3 ± 4.4 in the presence of pluronic; Fig. 2(c,d)). These results indicate that complete calibration measurements of F, F_min_, and F_max_ are required to estimate relative Zn^2+^ levels using FluoZin-3. We also determined that the addition of pluronic had only minor effects on the ratio F/F_min_ for both SpiroZin2 (ratio = 1.3 ± 0.17, − pluronic; 1.2 ± 0.17, + pluronic, *p* < 0.0001, Student’s *t*-test) and FluoZin-3 (ratio = 6.5 ± 4.6, − pluronic; 7.2 ± 4.6, + pluronic, *p* < 0.05, Student’s *t*-test). These data suggest that, although pluronic F-127 may affect the absolute amount of sensor taken up by a given cell, it does not affect sensor performance inside the cell.

When estimating relative labile Zn^2+^ concentrations in different cell populations, our experiments demonstrate the need for using normalized signals (F/F_min_), which give uniform and consistent results from cell to cell, rather than resting fluorescence intensities (F), which we found to be highly variable. A quantitative measure of variability is the coefficient of variation (CV). The CV of F for SpiroZin2 was 20.3–36.8%, whereas the CV of F/F_min_ was 12.4–17.4%. On the other hand, FluoZin-3 produced highly variable F (CV = 58.8–86.3%) and F/F_min_ (CV = 55.9–74.6%) results. Thus, when FluoZin-3 is used to estimate or compare intracellular labile Zn^2+^ levels under different conditions or in different samples, a large number of cells must be investigated to compensate for the signal variability at the single cell level.

Both SpiroZin2 and FluoZin-3 co-localize with lysosomal markers in a variety of eukaryotic cells including HeLa cells, cultured hippocampal neurons, breast cancer cells, and *C. elegans* intestinal cells^9,23,31,32^. FluoZin-3 was also reported to accumulate in the Golgi apparatus of HeLa cells and DT40 chicken B cells^25,33^. The mechanism by which dyes are sequestered in acidic vesicular compartments is not yet fully understood, and it is possible that a given dye can be distributed unequally across different types of vesicles (e.g. lysosomal, secretory, and Golgi apparatus vesicles). To study the distribution of SpiroZin2 and FluoZin-3 in different vesicular compartments, we performed a colocalization analysis of each dye with three vesicular markers, LAMP1-EBFP, GalT-CFP, and VAMP8-CFP. Lysosome associated membrane protein 1 (LAMP1) is a highly glycosylated integral membrane protein that is found in late endosomes and lysosomes, and it is frequently used as a marker for these organelles^34^. GalT-CFP comprises the first sixty amino acids of human galactosyltransferase (GalT), a trans-Golgi targeting sequence, fused to CFP. Vesicle-associated membrane protein 8 (VAMP8) is a marker for secretory vesicles that is associated with exocytosis in different types of mammalian cells including pancreatic acinar cells^35^, mast cells^36^, cytotoxic T lymphocytes^37^, platelets^38^, and kidney collecting duct epithelia^39^.

**Figure 3:**
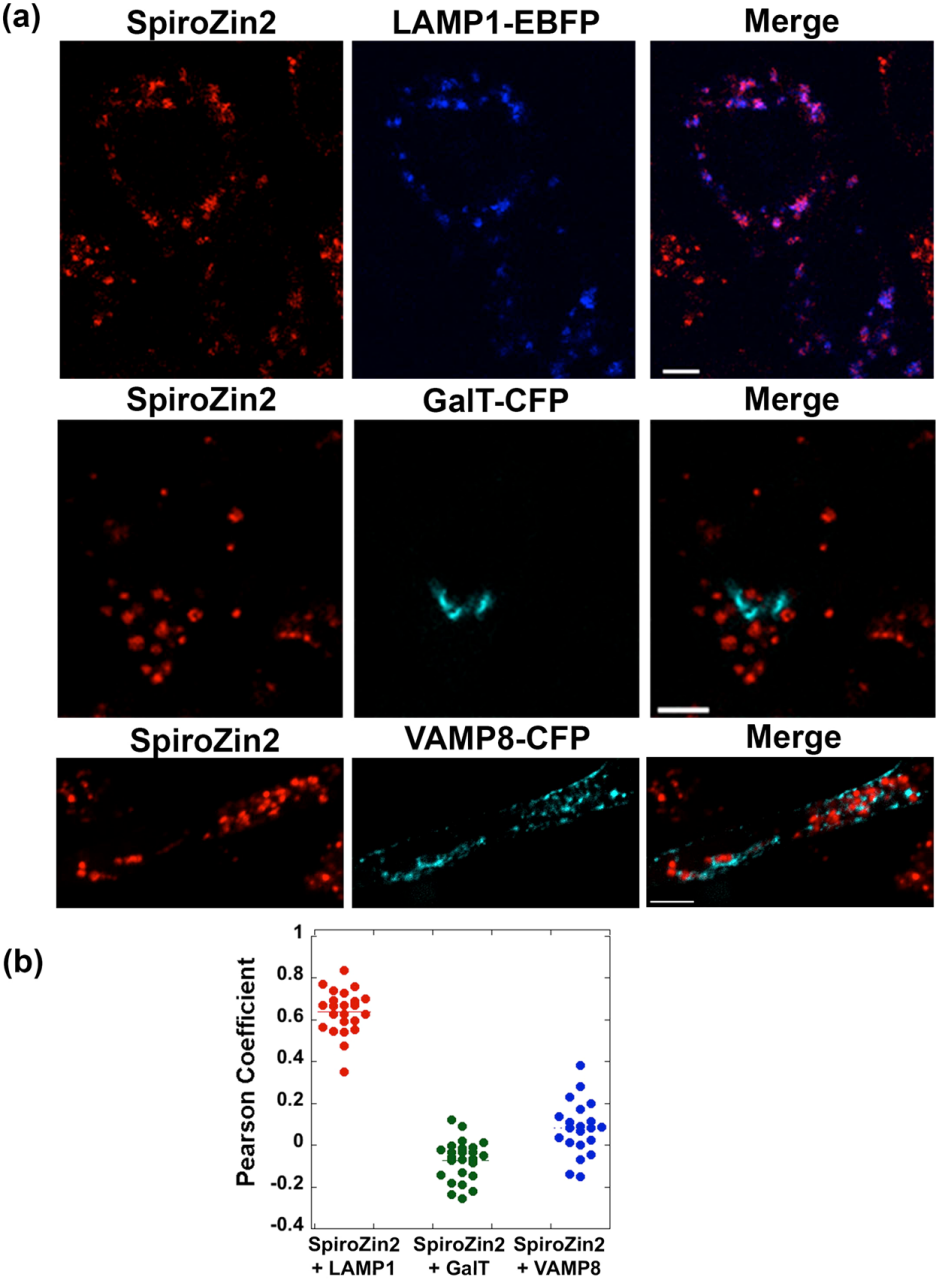
Co-localization analysis of SpiroZin2 and three vesicular markers demonstrates that SpiroZin2 resides in lysosomal vesicles. (**a**) Representative images of SpiroZin2-stained cells, vesicular markers- FP, and merged channel. (**b**) Pearson coefficient plot of co-localization analysis. Pearson analysis included a minimum of 21 cells per condition. Scale bar, 5 μm.

**Figure 4:**
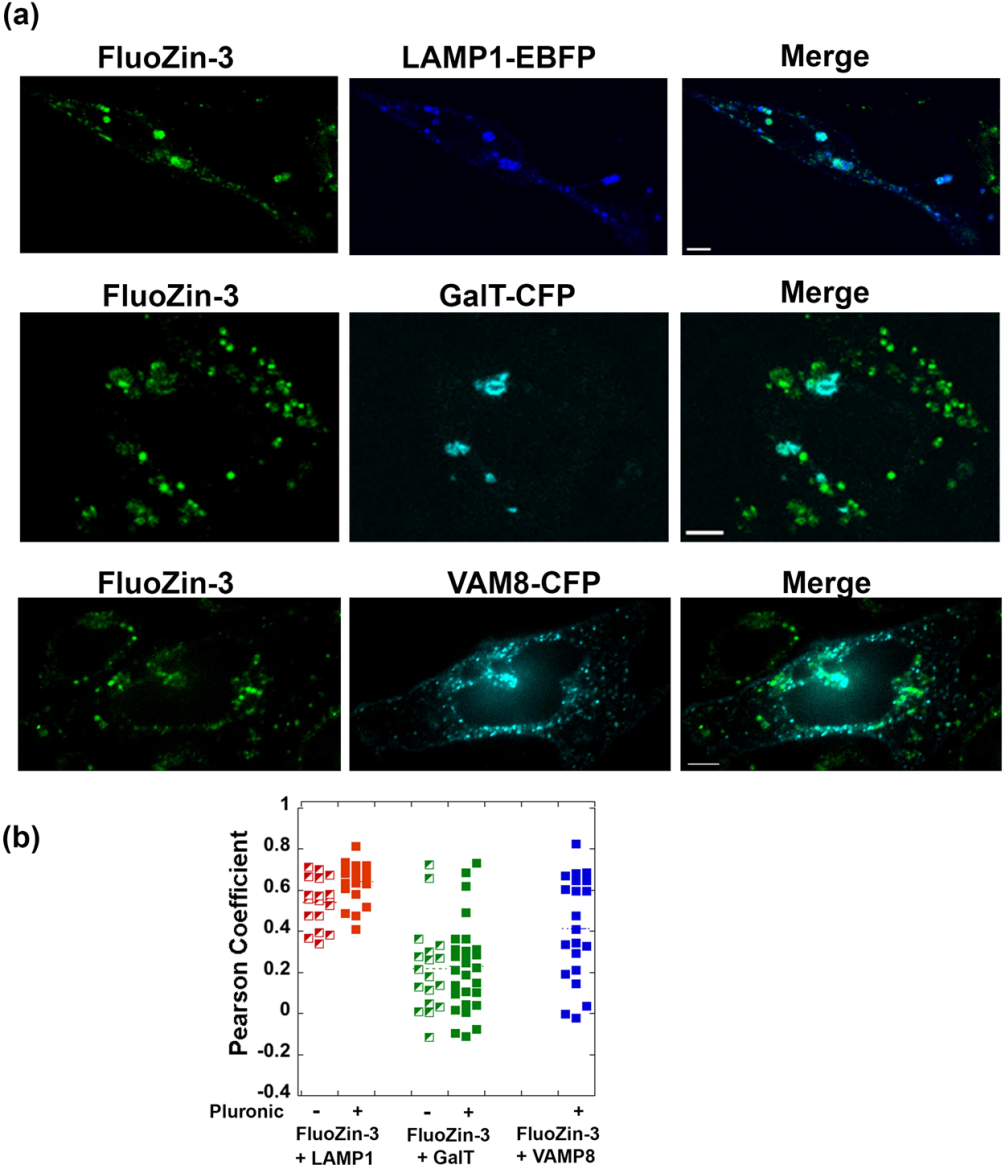
Co-localization analysis of FluoZin-3 and three vesicular markers demonstrates that FluoZin-3 non-specifically localizes to lysosomal, Golgi, and secretory vesicles. (**a**) Representative fluorescence images of Spirozin2-stained cells, vesicular markers- FP, and the merged channel. (**b**) Pearson coefficient plot of co-localization analysis. A minimum of 18 cells were analyzed for each condition. Scale bar, 5 μm.

To perform the co-localization assay, HC11 cells transiently expressing FP-tagged vesicular markers were stained with SpiroZin2 or FluoZin-3 and imaged by fluorescence microscopy. Dual color images were analyzed with an intensity correlation assay based on the Pearson correlation coefficient (PC), which ranges from −1 to 1, with −1 indicating negative correlation, 0 no correlation, and 1 complete positive correlation. PC analysis requires the intensities of the two channels to be comparable because uneven intensities of two channels can lead to artificially diminished PC values^40^. Under resting conditions in HC11 cells, the fluorescence intensities of the sensors were too low compared to those of the FP-tagged marker proteins, presumably due to the low levels of intracellular Zn^2+^. In order to achieve comparable intensities in the dye and marker-FP channels, we added a Zn^2+^/pyrithione solution to dye-stained cells, which increased the sensor fluorescence intensities after a few minutes. PC-based co-localization analysis revealed that SpiroZin2 was strongly co-localized with LAMP1 (PC = 0.68 ± 0.11), but poorly co-localized with GalT (PC = −0.07 ± 0.10) and VAMP8 (PC = 0.08 ± 0.13), indicating that it preferentially resides in late endosomes and lysosomes (Fig. 3). Because FluoZin-3 is commonly delivered to cells with pluronic, we tested whether this procedure affects subcellular localization. Figure 4 shows that FluoZin-3 co-localizes with LAMP1 reasonably well (PC = 0.54 ± 0.12 without pluronic, 0.64 ± 0.1 with pluronic). However, we observed that some portion of FluoZin-3 also populates the Golgi (PC = 0.22 ± 0.22 without pluronic, 0.23 ± 0.21 with pluronic) and secretory vesicles (PC = 0.41 ± 0.25 with pluronic). Because vesicular compartments may have different amounts of labile Zn^2+^, the non-specific localization of FluoZin-3 may contribute to the highly variable fluorescence intensities of FluoZin-3 staining in Fig. 2.

**Figure 5:**
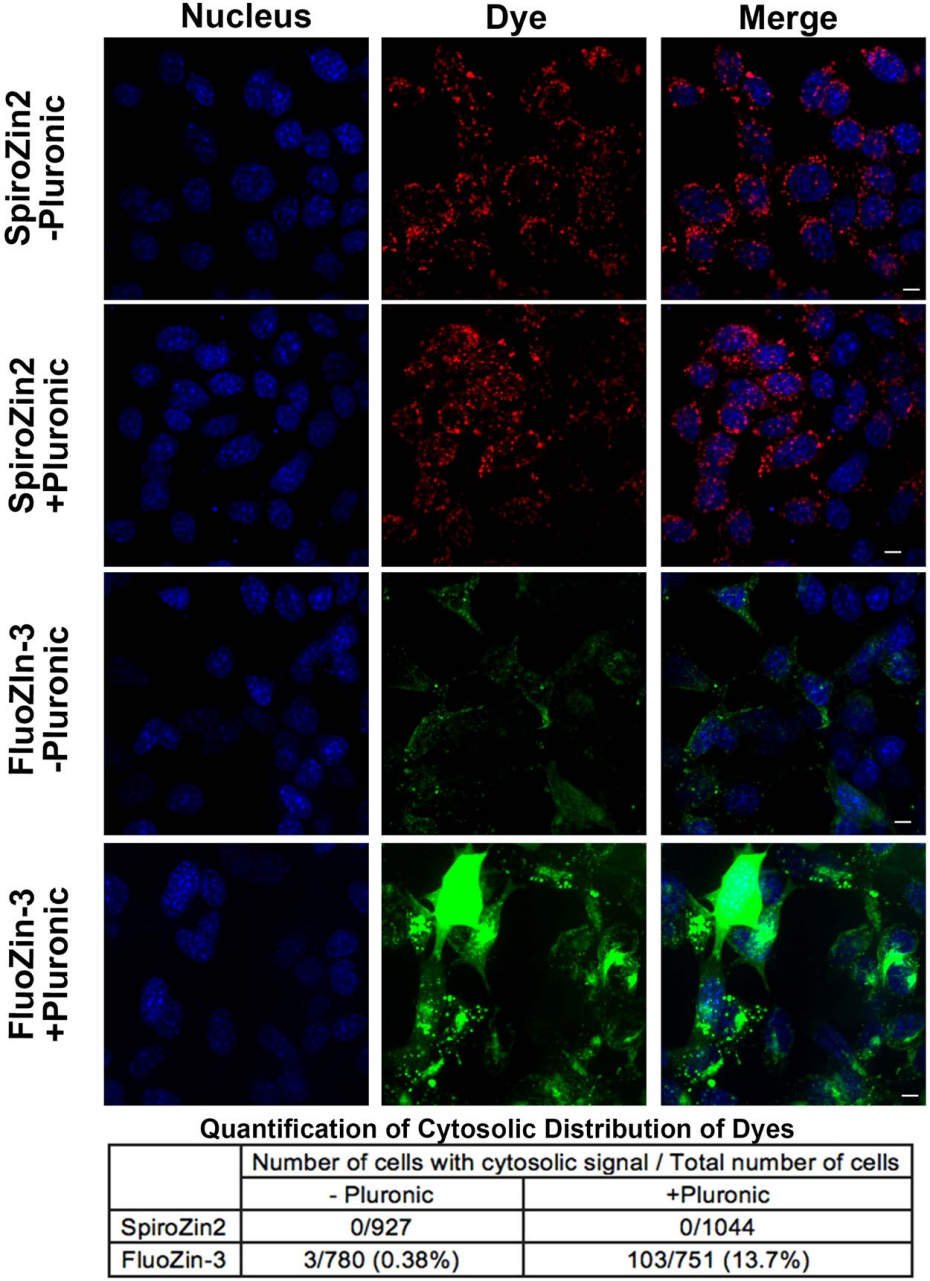
A population of FluoZin-3 localizes to the cytosol. HC11 cells were stained with SpiroZin2 or FluoZin-3 with and without pluronic. Fluorescence images in each channel were acquired 5 min post treatment with 5 μM pyrithione/20 μM ZnCl_2_. Quantification reveals that SpiroZin2 does not localize to the cytosol but the cytosolic localization of FluoZin-3 depends on the presence of pluronic. Scale bar, 5 μm.

Similar to reports from other researchers^9,25^, we observed cytosolic signals in some cells treated with FluoZin-3 (Fig. 1(d)). To rigorously quantify the cytosolic distribution of SpiroZin2 and FluoZin-3, we stained HC11 cells with each dye in the presence and absence of pluronic and then determined the percentage of cells exhibiting cytosolic signals. As shown in Fig. 5, none of the SpiroZin2-stained cells showed cytosolic signals under any condition, whereas 0.38% and 13.7% of FluoZin-3-treated cells exhibited cytosolic staining in the absence and presence of pluronic, respectively. In the presence of pluronic, the increase in cytosolic FluoZin-3 signals may be attributable to the increased aqueous solubility of FluoZin-3 AM. Alternatively, it is possible that pluronic facilitates the general uptake of FluoZin-3 into cells and the dye accumulates in cytosol after saturating vesicular organelles. Therefore, when using pluronic it is advisable to optimize the concentration of the FluoZin-3 to avoid accumulation of dye in the cytoplasm. In contrast, SpiroZin2 exhibited unambiguous and consistent vesicular localization.

**Figure 6:**
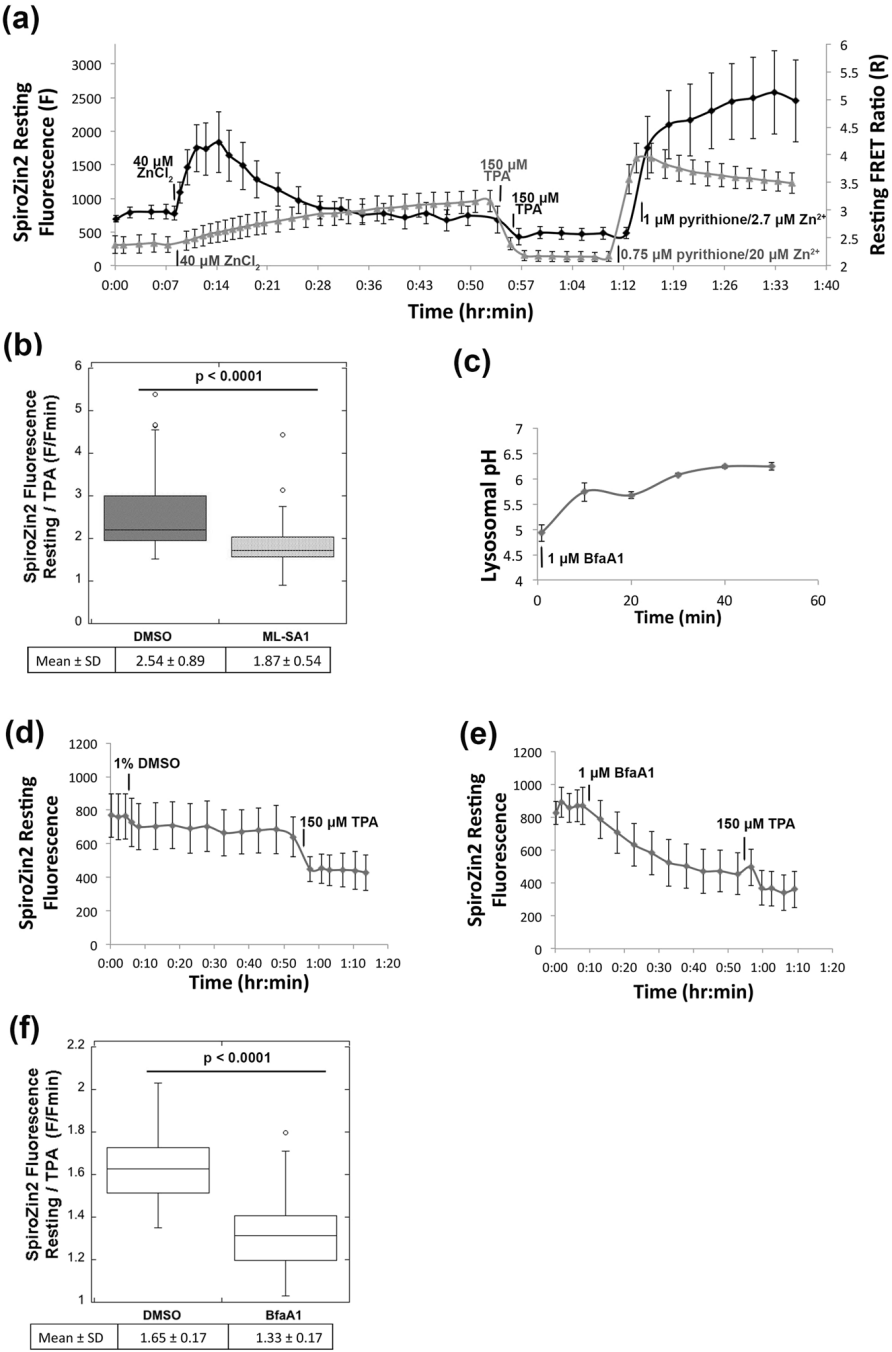
SpiroZin2 reveals changes in lysosomal Zn^2+^ upon perturbation. (**a**) The resting fluorescence of SpiroZin2 (black trace, left y axis) and resting FRET ratio (gray trace, right y axis) of ZapCV2 were recorded over time following treatment with 40 μM ZnCl_2_, 150 μM TPA and 1 μM pyrithione/2.7 μM Zn^2+^ (SpiroZin2), 0.75 μM pyrithione/20 μM Zn^2+^ (ZapCV2). (**b**) Normalized SpiroZin2 fluorescence signal (F/F_min_) decreased upon treatment with ML-SA1 compared to the vehicle (DMSO), suggesting a decrease in lysosomal Zn^2+^ upon ML-SA1 treatment. F, SpiroZin2 fluorescence at 30 min post ML-SA1 or DMSO treatment. F_min_, SpiroZin2 fluorescence at 12 min post TPA treatment. n > 52, *p* < 0.0001, unpaired student’s t-test. (**c**) Lysosomal pH increased upon treatment with 1 μM bafilomycin A1. SpiroZin2 resting fluorescence was recorded every 5 min for 45 min post treatment with vehicle (1% DMSO) (**d**) or 1 μM bafilomycin A1 (**e**), followed by treatment with 150 μM TPA. (**f**) Normalized SpiroZin2 fluorescence (F/F_min_) in bafilomycin A1-treated cells is lower than that in DMSO-treated cells. F, the mean of the fluorescence intensities at 40 min and 45 min post treatment with DMSO or bafilomycin A1; F_min_, the mean of the fluorescence intensities of the last three time points post TPA treatment. n > 24, *p* < 0.0001, unpaired student’s t-test.

To further characterize the ability of SpiroZin2 to detect lysosomal Zn^2+^, we investigated the dynamic range of the sensor in lysosomes and monitored the lysosomal Zn^2+^ pool as a function of a series of perturbations. To define the full dynamic range, we carried out *in situ* calibrations using TPA to determine F_min_ and Zn^2+^ with pyrithione to determine F_max_. As shown in Fig. 6(a), the dynamic range of SpiroZin2 in lysosomes is 5.3 ± 1.2. An important feature of a lysosomal probe is insensitivity to pH. Whereas SpiroZin2 is insensitive to pH changes between 4.0 and 6.0 *in vitro*, this property was not previously tested in cells. To assess the pH dependence of SpiroZin2 in cells, we first validated that LysoSensor^TM^ Yellow/Blue Dextran, a lysosomal pH indicator, could detect changes in pH from 4 to 6 (Supplementary Fig. 1(a)). We demonstrated that treatment of cells with NH_4_^+^ lead to a lysosomal pH shift within 2.5 min (Supplementary Fig. 1(b)) and showed that this shift in pH did not alter SpiroZin2 fluorescence intensity (Supplementary Fig. 1(c)), confirming that SpiroZin2 fluorescence is not affected by changes in pH within this range in cells.

We also examined how lysosomal Zn^2+^ pools respond to an increase in extracellular Zn^2+^. Previously we demonstrated that elevation of extracellular Zn^2+^ leads to a slow increase in cytosolic Zn^2+^ in HeLa cells followed by sequestration into intracellular organelles such as the ER and mitochondria^14^. Here, we show that HC11 cells also exhibit a steady increase in cytosolic Zn^2+^ detected via the genetically encoded fluorescent Zn^2+^ sensor ZapCV2^41^, when treated with 40 μM extracellular Zn^2+^ (Fig. 6(a). Surprisingly, lysosomal Zn^2+^ increased more quickly than cytosolic Zn^2+^, reaching a peak within 5 min, while cytosolic Zn^2+^ continued to rise after 45 min of treatment. This result suggests that the extracellular Zn^2+^ is rapidly internalized into lysosomes via endocytosis, which operates on a time scale of seconds to minutes^42^. Our data also revealed that this increase in lysosomal Zn^2+^ was transient; the Zn^2+^ level dropped quickly and stabilized within 20 min. The possible routes of lysosomal Zn^2+^ export could be Zn^2+^ leak through lysosomal ion channel transient receptor potential mucolipin 1 (TRPML1)^43^, and/or export to the extracellular environment by lysosomal exocytosis^44^. To further validate that SpiroZin2 specifically monitors lysosomal Zn^2+^, we treated cells with ML-SA1, a TRPML1 agonist^45^ that activates Ca^2+^ release from lysosomes^46,47^. We discovered that ML-SA1 treatment led to a decrease in normalized SpiroZin2 fluorescence compared to vehicle (DMSO) treatment (Fig. 6(b)), consistent with reported studies that TRPML1 mediates a Zn^2+^ leak from lysosomes in human and mouse cells^10,43^. Together, these data demonstrate that SpiroZin2 is capable of monitoring changes in lysosomal Zn^2+^ level upon direct modulation of lysosome Zn^2+^ transport.

Finally, we used bafilomycin A1, a vacuolar H^+^ ATPase inhibitor,^48^ to dissipate the H^+^ gradient across the lysosomal membrane (Fig. 6(c)), and show that this procedure decreases the lysosomal Zn^2+^ pool, as detected by a decrease in normalized SpiroZin2 fluorescence intensity (Fig. 6(d–f)). These results suggest that lysosomal Zn^2+^ accumulation is facilitated by the proton gradient via Zn^2+^/H^+^ exchange. The Cation Diffusion Facilitator (CDF) protein family transports divalent cations and is present in diverse organisms from bacteria to humans^49^. It is well established that the *E.coli* CDF protein YiiP acts as a Zn^2+^/H^+^ antiporter^50,51^. YiiP is a homologue of mammalian ZnT transporters, and Zn^2+^ transport by ZnT1 and ZnT5 has been suggested to be facilitated by proton gradients^52,53^. Given that ZnT2 and ZnT4 transport Zn^2+^ into lysosome^43,54,55^, it is possible that ZnT2 and ZnT4 act as Zn^2+^/H^+^ antiporters, whereby dissipation of the proton gradient disrupts lysosomal Zn^2+^ accumulation.

**Figure 7:**
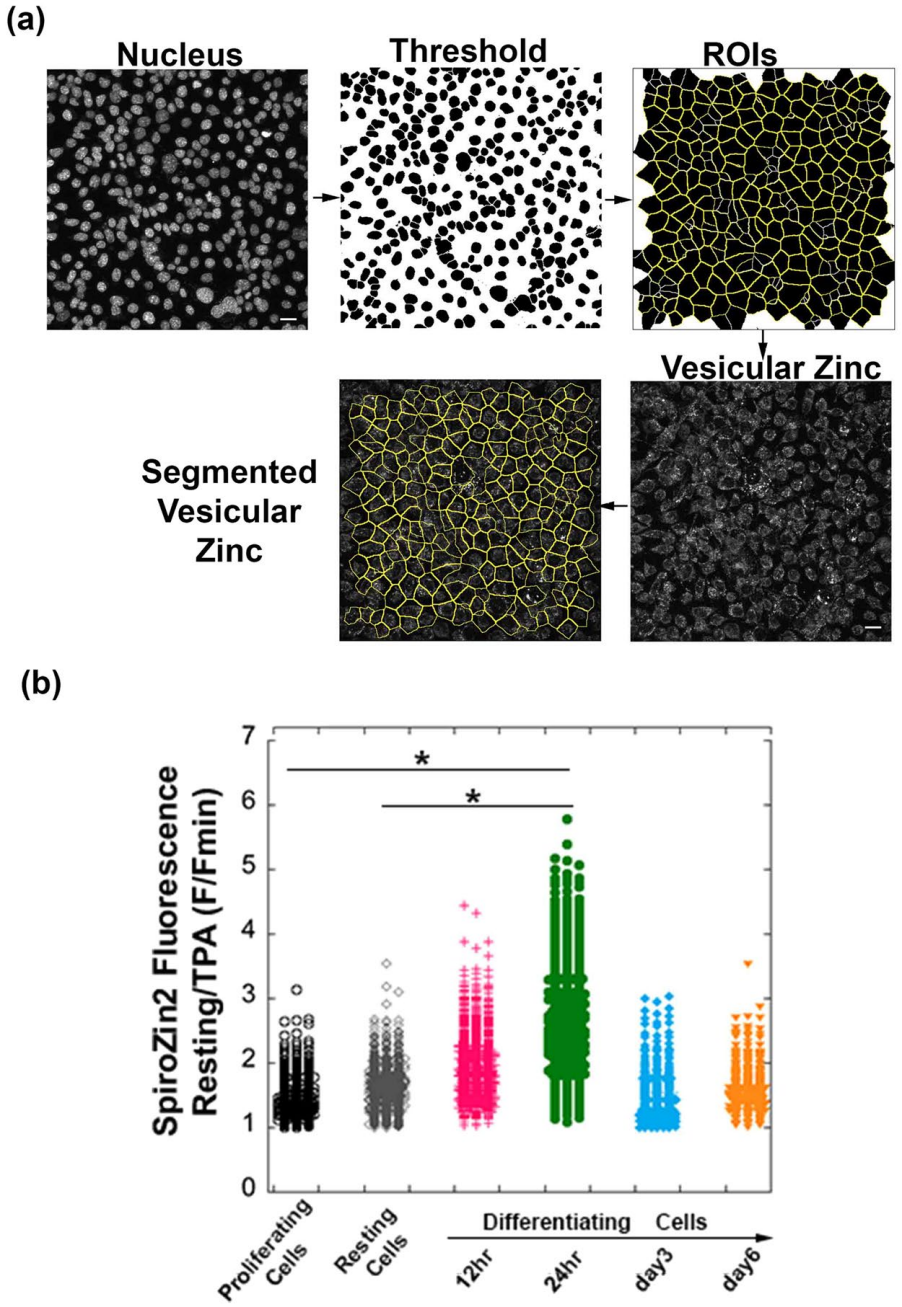
A substantial increase in lysosomal Zn^2+^ was detected in SpiroZin2-stained mammary epithelial cells at 24 hr post hormone treatment (5 μg/mL insulin, 5 μg/mL prolactin, and 1 μM hydrocortisone). (**a**) Cell segmentation method for single-cell analysis. Scale bar, 40 μm. (**b**) SpiroZin2 fluorescence signal at resting state normalized to its signal after 30 min of TPA treatment. Each point is an individual cell, n > 297 for each sample. Two biological replicates per condition. ^*^p < 0.0001, one-way ANOVA test.

Given its superior performance, we used SpiroZin2 to track vesicular Zn^2+^ concentrations in mouse mammary epithelial cells (HC11), which differentiate in response to lactation hormones. Because accumulation of Zn^2+^ in intracellular vesicles in secreting HC11 cells has been hypothesized to be a hormone-dependent process^13^, we chose to study this phenomenon. Previous reports indicate that lysosome-related organelles in *C. elegans* intestinal cells serve as major Zn^2+^ storage sites that play important roles in maintaining Zn^2+^ homeostasis^32^. To examine whether late endosomes/lysosomes serve a similar function in secreting HC11 cells, we induced differentiation of HC11 cells with lactation hormones according to established protocols^56,57^ and analyzed the relative Zn^2+^ concentrations by SpiroZin2 as a function of time. Single-cell analyses were performed as illustrated in Fig. 7(a). Cells were stained simultaneously with a nuclear dye and SpiroZin2. Nucleus channel images were analyzed with ImageJ to identify the nuclear area, segment cells, and define regions of interest (ROIs), with each ROI corresponding to a single cell. The mean fluorescence intensity in the corresponding SpiroZin2 channel was quantified for each cell in the entire field of view. Normalized fluorescence intensities were measured by treating the cells with TPA and calculating F/F_min_, as described above. As shown in Fig. 7(b), we detected a substantial increase in labile Zn^2+^ concentrations 24 hours after hormone treatment relative to proliferating resting cells (*p* < 0.0001), an observation that suggests late endosomes or lysosomes may serve as temporary storage sites for Zn^2+^ during HC11 cell differentiation.

## Discussion

Live-cell imaging of individual cells using small-molecule fluorescent probes is a powerful and widely used approach for estimating the concentration and distribution of analytes in cells. However, few studies rigorously evaluate the heterogeneity in fluorescence signal and localization from cell to cell and explicitly compare the functionality of different probes. In this study, we performed a parallel and systematic comparison of two small-molecule vesicular Zn^2+^ probes, FluoZin-3 AM and SpiroZin2, to evaluate each probe for measurement of vesicular Zn^2+^ pools. Our work reveals that SpiroZin2 specifically reports on lysosomal Zn^2+^ pools and can be used to monitor changes in lysosomal Zn^2+^ in response to a variety of perturbations (changes in extracellular Zn^2+^, activation of TRPML1, dissipation of the lysosomal H^+^ gradient, and cell differentiation).

One of the primary applications of small molecule fluorescent Zn^2+^ sensors is to compare relative amounts of Zn^2+^ in different biological samples, for example between different cell types or between cells in different physiological states, such as differentiated versus undifferentiated. As is evident in published studies^26–30^, it is common practice to use resting fluorescence intensities of FluoZin-3 to estimate relative Zn^2+^ levels in different cell populations. Our results indicate that the resting FluoZin-3 or SpiroZin2 fluorescence intensities (F) are highly variable among cells (Fig. 2), with an average coefficient of variation (CV) of 28% for SpiroZin2 and 73% for FluoZin-3, and should not be used to represent relative Zn^2+^ levels. In contrast, for SpiroZin2 the ratio of fluorescence intensity of the resting state to that after TPA treatment (F/F_min_) was uniform and consistent, with a substantially decreased average CV (13.7%, Fig. 2). Therefore, instead of F, F/F_min_ is a more appropriate and precise parameter to assess relative labile Zn^2+^ levels in cells when using SpiroZin2. Unfortunately for FluoZin-3, fluorescence signals normalized to F_min_ (F/F_min_) remained highly variable (average CV 66%). One possible explanation for this high variability in fluorescence signal under resting conditions is that individual cells internalize different amounts of dye. In this case, normalization of F to the F_min_ signal in each individual cell provides an internal control for that cell and mitigates the variability. For FluoZin-3, the large degree of signal heterogeneity, even after normalization, most likely results from the widespread heterogeneity in dye localization in addition to affects from differential dye accumulation.

Unlike targeted genetically-encoded sensors that can be localized to a specific organelle by a tagging sequence, small molecule fluorescent probes tend to accumulate adventitiously in different compartments^25^. Because previous reports suggest that FluoZin-3 AM accumulates in multiple organelles in mammalian cells^58^, we rigorously determined the intracellular distribution of the dyes. Our data reveal that SpiroZin2 exclusively localizes in lysosomal vesicles. Whereas previous work^23^ showed SpiroZin2 accumulated in lysosomes, it did not establish that SpiroZin2 was exclusively in lysosomes, as we do here. FluoZin-3 AM non-specifically accumulates in the cytosol, as well as across three populations of vesicles: lysosomal vesicles, trans-Golgi vesicles and secretory vesicles. Owing to possible different labile Zn^2+^ levels in different vesicular compartments, the heterogeneous intracellular localization of FluoZin-3 may contribute to the variable fluorescence intensities of FluoZin-3 staining observed in Fig. 2, even after normalization to F_min_. This high degree of variation confounds interpretation of Zn^2+^ measurements made by FluoZin-3 in different biological samples or under different conditions. In order to compare relative Zn^2+^ levels in a particular subcellular compartment across samples or conditions using FluoZin-3, researchers should simultaneously measure FluoZin-3 signals and organelle markers, carry out full probe calibrations by recording F, F_min_, and F_max_ in individual cells, and restrict analysis to those cells in which FluoZin-3 co-localizes with a genetically-encoded marker for the compartment of interest.

In the present study we used F/F_min_ to provide a relative measure of the labile Zn^2+^ pool in lysosomes. It should be noted that, although sensors can be used to quantify Zn^2+^, doing so requires a full sensor calibration, estimation of the dissociation constant (K_d_) for Zn^2+^ ideally in the compartment of interest, and demonstration that the probe is in equilibrium with the labile pool within a particular compartment. Small compartments, such as lysosomes, pose an additional challenge because of their extremely small volumes.

Our results reveal that SpiroZin2 is a specific lysosomal Zn^2+^ probe that provides reliable measurements of resting lysosomal Zn^2+^ pools in single cell analyses, when properly normalized, and how these pools change with different cellular perturbations. We demonstrate that lysosomal Zn^2+^ pools respond rapidly to perturbation of extracellular Zn^2+^. We further show that the lysosomal Zn^2+^ pool decreases upon activation of the Zn^2+^-permeable channel TRPML1 and relies on activity of the V-ATPase of lysosomes. We also applied SpiroZin2 to lactating mouse mammary epithelial cells and detected a transient increase of lysosomal free Zn^2+^ 24 hrs after treatment with a lactation hormone. This finding is significant because a growing number of studies suggest that lysosomes play a key role in Zn^2+^ homeostasis. Zn^2+^ can be delivered into lysosomes from the cytosol by two Zn^2+^ transporters, Slc30a2 and Slc30a4^43,54,55^, or through other cellular processes such as autophagy^59,60^. Lysosomes serve as Zn^2+^ storage reserves and protect cells from Zn^2+^ overload in *C. elegans* intestinal cells and HeLa cells^32,44^. Even so, lysosomal accumulation of Zn^2+^ can also be toxic, inducing lysosomal membrane permeabilization (LMP) and cell death under certain pathological conditions^9,24,61,62^. Lysosomal Zn^2+^ can be transported into the cytosol by TRPML1^43^ and/or exported to the extracellular environment by lysosomal exocytosis^44^. Although the mechanism of the transient increase of lysosomal Zn^2+^ lactating mouse mammary epithelial cells is unknown, we propose that lysosomes serve as temporary Zn^2+^ storage sites and play key roles in the regulation of Zn^2+^ homeostasis during lactation. Previous work has implicated lysosomes as playing an important role in redistribution of Zn^2+^ pools during involution of mammary tissue upon weaning^63–65^. Here, we show they also play a role during differentiation to a lactation phenotype, suggesting that lysosomes may be a major player during remodeling of Zn^2+^ homeostasis and redistribution of labile Zn^2+^ pools.

## Methods

### Chemicals

SpiroZin2 was synthesized as previously described and a 4 mM stock solution was prepared in DMSO^23^. Tris(2-pyridylmethyl)amine (TPA) was purchased from Sigma-Aldrich (catalog number 723134) and diluted with DMSO to prepare 20 mM stock solutions. Pluronic F-127 (20% (w/v) solution in DMSO) was purchased from Thermo Fisher (catalog number P3000MP). FluoZin-3 AM (Thermo Fisher, catalog number F24195) was prepared as a 2 mM stock solution in DMSO. Stock solutions (5 mM) of 2-mercaptopyridine *N*-oxide (pyrithione, Sigma-Aldrich, catalog number 188549) were prepared in DMSO. Nucleus staining was achieved with NucBlue Live ReadyProbes Reagent (Thermo Fisher, catalog number R37605). An aqueous ZnCl_2_ solution (1 mM) was diluted in phosphate-free HEPES-buffered Hanks’ balanced salt (HHBSS) buffer (1.26 mM CaCl_2_, 1.1 mM MgCl_2_, 5.36 mM KCl, 137 mM NaCl, 16.65 mM D-glucose, and 30 mM HEPES, pH 7.4) to make a 200 μM ZnCl_2_ stock solution. An insulin stock solution (4 mg/mL) was purchased from Life Technologies (catalog number 12585014). Human recombinant epidermal growth factor (EGF, VWR 47743-566) was prepared as a 10 μg/mL stock solution in water. Prolactin (Sigma-Aldrich L6520) was prepared as a 5 mg/mL stock solution in water. A stock solution (138 μM) of hydrocortisone (Sigma-Aldrich H0888) in absolute ethanol was also prepared. ML-SA1 (Abcam ab144662) was prepared as a 50 mM stock solution in DMSO. Bafilomycin A1 (Sigma-Aldrich B1793) was prepared as a 100 μM stock solution in DMSO.

### Molecular Cloning

The GalT-CFP plasmid encodes the first 60 amino acids of human galactosyltransferase (GalT) (NM_001497.3) followed by ECFP in the Clontech N1 vector. VAMP8-CFP was generated by replacing GalT with VAMP8 (NM_003761.4). LAMP1-EBFP was a gift from Michael Davidson (Addgene # 55246).

### Cell Culture

HC11 cells were grown in RPMI medium supplemented with 10% fetal bovine serum (FBS), 1% penicillin/streptomycin (pen/strep), 10 ng/mL EGF and 5 μg/mL insulin. To induce cell differentiation, 4 × 10^5^ cells were plated in a 35-mm dish in the supplemented medium and reached confluency after 3 days. Cells were maintained in a resting medium containing RPMI, 2% FBS, 1% pen/strep, 5 μg/mL insulin for one additional day, then treated with a differentiation medium containing 2% FBS, 1% pen/strep, 5 μg/mL insulin, 5 μg/mL prolactin, and 1 μM hydrocortisone for up to 6 days. The medium was changed every 2 days.

### Cell Loading with Zinc Probes and Calibration

FluoZin-3 AM and SpiroZin2 were loaded as previously described according to manufacturer's instructions^23^. Briefly, a 2 mM stock solution of FluoZin-3 AM DMSO stock solution was diluted to a final concentration of 3 μM in phosphate-free HHBSS buffer. Phosphate-free HHBSS buffer was used to avoid precipitation of Zn^2+^ by phosphate. HC11 cells were washed with phosphate-free HHBSS and then incubated with FluoZin-3 AM/phosphate-free HHBSS for 1 h at 37 °C with 5% CO_2_, followed by a 30 min wash in phosphate-free HHBSS buffer at 37 °C with 5% CO_2_. Imaging was performed immediately after washing. For SpiroZin2, a 4 mM DMSO stock solution was diluted to a final concentration of 5 μM in phosphate-free HHBSS buffer. HC11 cells were washed with phosphate-free HHBSS buffer and then incubated with 2 mL of SpiroZin2 solution for 1 h at 37 °C with 5% CO_2_ prior to imaging. To apply pluronic during the staining procedure, the stock solution of zinc probe was mixed with an equal volume of Pluronic F-127 20% (w/v) solution and then the mixture was diluted in phosphate-free HHBSS buffer, making the final pluronic concentration about 0.025%. For Zn^2+^ calibrations, F_min_ measurements were acquired by treating cells with 150 μM TPA in phosphate-free HHBSS buffer. To obtain values for F_max_, a solution containing 5 μM pyrithione/20 μM ZnCl_2_ in phosphate-free HHBSS buffer was added to HC11 cells.

### Live Cell Imaging

Fluorescence images in Figs. 1–3 and 5 were acquired on a Nikon A1R laser scanning confocal microscope equipped with the Nikon Elements software platform, Ti-E Perfect Focus system with a Ti Z drive, using a 40x oil objective (NA 1.30) or a 100x oil objective (NA 1.45) and the following channels: SpiroZin2 (red) (514.5 nm laser line, photomultiplier tube (PMT) gain: 50 or 110, pinhole size: 1 or 4 airy unit (AU), emission filter: 600/50 nm); FluoZin-3 (green) (488 nm laser line, PMT gain: 45 or 110, pinhole size: 1 or 4 AU, emission filter: 525/50 nm); nucleus (blue) (405 nm laser line, PMT gain: 80 or 95, pinhole size: 1 AU, emission filter: 450/50 nm); LAMP1-EBFP (blue) (405 nm laser line, PMT gain: 95 or 110, pinhole size: 1 or 4 AU, emission filter: 450/50 nm); GalT-CFP (Cyan) (405 nm laser line, PMT gain: 90, pinhole size: 4 AU, emission filter: 482/35 nm).

Fluorescence images for colocalization of fluorescent probes with VAMP8-CFP in Fig. 3 and all fluorescence images in Fig. 4 were acquired on a Nikon Ti-E spinning disc confocal microscope fitted with a Yokogawa CSU-X1 spinning disc head, equipped with the Nikon Elements software platform and Ti-E Perfect Focus system. Cells were imaged using a 60x oil objective (NA 1.40) or a 100x oil objective (NA 1.45) and the following channels: SpiroZin2 (red) (514 nm laser line, EM gain: 300, emission filter: 630/30 nm), FluoZin-3 (green) (488 nm laser line, EM gain: 300, emission filter: 525/50 nm), Vamp8-CFP (cyan) (445 nm laser line, EM gain: 300, emission filter: 482/35 nm).

Fluorescence imaging with a Zn^2+^ FRET sensor was performed on a Nikon Ti-E wide-field fluorescence microscope equipped with Nikon elements software, an iXon3 EMCCD camera (Andor), mercury arc lamp, and YFP FRET (434/16 excitation, 458 dichroic, 535/20 emission), CFP (434/16 excitation, 458 dichroic, 470/24 emission), YFP (495/10 excitation, 515 dichroic, 535/20 emission) filter sets.

### Responses of Probes to Zn^2+^ Perturbation in HC11 Cells

To test the responses of probes to Zn^2+^ perturbation, 8 × 10^5^ HC11 cells were plated in a 35-mm glass bottom dish one day prior to imaging. Cells were stained with FluoZin-3 AM (with pluronic) or SpiroZin2 as described above. The nuclear dye was added to the cell culture medium 30 min prior to imaging. Cells were washed with phosphate-free HHBSS buffer and then imaged on a Nikon A1R laser scanning confocal microscope as described in the “Live Cell Imaging” section. Z-series images were recorded every 3 min throughout the experiment. After adding Zn^2+^/pyrithione to cells, PMT gain was lowered from 50 to 15 for SpiroZin2 and from 45 to 25 for FluoZin-3 to avoid detector saturation. Image analysis was performed using ImageJ. Z-series images of each fluorescence channel per time point were flattened using the maximum intensity Z-projection. To perform single cell analyses, 15 cells were randomly chosen and each cell was defined as an individual region of interest (ROI). Background fluorescence of each channel was calculated by averaging the signal intensities of an area (mean intensities) without cells. The fluorescence intensities of the FluoZin-3 or SpiroZin2 channel of each ROI were calculated by subtracting background fluorescence from the raw mean fluorescence intensities for each channel.

### Variability of Fluorescence Intensities of Zinc Probes

To test the variability of fluorescence intensities of FluoZin-3 and SpiroZin2, 8 × 10^5^ HC11 cells were plated in a 35-mm glass bottom dish one day prior to imaging. Cells were stained with FluoZin-3 or SpiroZin2 with or without pluronic as described above. Nuclear dye was added to the cell culture medium 30 min prior to imaging. Cells were washed with phosphate-free HHBSS buffer and then imaged on a Nikon A1R laser scanning confocal microscope as described in the “Live Cell Imaging” section. Z-series images were recorded in the resting state (F) and 15 min post TPA treatment (F_min_). Image analysis was performed using ImageJ. Z-series images of each fluorescence channel per time point were flattened using the maximum intensity Z-projection. To perform single cell analyses, nucleus channel images were thresholded to identify the nucleus area. Touching objects were separated by a watershed algorithm. Next, segmented particles were identified by the “find maxima” command with “segmented particles” as the output type, and “light background” chosen when the background color was white. To create ROIs with each ROI corresponding to a single cell, segmented pictures were processed by the “analyze particles” command as follows: the range of the size of particles was adjusted so that each particle incorporated a single cell and then the option “add to manager” was chosen to generate a list of ROIs. Applying the ROIs to the FluoZin-3 or Spirozin2 channel images allowed the quantification of the mean raw intensity of the sensor fluorescence at the single-cell level. The background fluorescence of each channel was calculated by averaging the signal intensities of an area (mean intensities) without cells. The fluorescence intensities of FluoZin-3 or SpiroZin2 channel of each cell were calculated by subtracting the background fluorescence from the raw mean fluorescence intensities.

### Colocalization Assay of Zinc Probes with Vesicular Markers

To transiently express vesicular markers in HC11 cells, 500 ng of DNA encoding each fusion protein (LAMP1-EBFP, VAMP8-CFP or GalT-CFP) was electroporated into 1 × 10^6^ cells with the Neon system (Life Technologies) 24–48 h prior to imaging using the following parameters: pulse voltage: 1400 V; pulse width: 20 ms; pulse number: 2 pulses. Electroporated cells were plated in 35-mm glass-bottom imaging dishes. Before imaging, cells were stained with FluoZin-3 or SpiroZin2 with or without pluronic as described above. After staining, cells were washed with phosphate-free HHBSS buffer. To enhance the fluorescence intensities of zinc probe channels, 5 μM pyrithione/20 μM ZnCl_2_ was added to cells. Fluorescence images were recorded 5–10 min after Zn^2+^ addition. Sensor co-localization experiments with LAMP1-EBFP and GalT-CFP were performed on a Nikon A1R laser scanning confocal microscope as described in the “Live Cell Imaging” section. Colocalization experiments with VAMP8-CFP were performed on a Nikon Ti-E spinning disc confocal microscope as described in the “Live Cell Imaging” section.

To perform the Pearson coefficient-based colocalization assay, cells with fluorescence signals in both the zinc sensor channel and the vesicular maker channel were selected for analysis with JACoP plugin for ImageJ (https://imagej.nih.gov/ij/plugins/track/jacop.html). An important criterion for choosing cells is that the fluorescence intensities of the two channels must be comparable. A region of interest (ROI) was drawn within a cell and the Pearson coefficient was calculated by JACoP. Data were plotted with KaleidaGraph.

### Cytosolic Distribution of Zinc Probes

To determine the cytosolic distribution of FluoZin-3 and SpiroZin2, 8 × 10^5^ HC11 cells were plated in a 35-mm glass bottom dish one day prior to imaging. Cells were stained with FluoZin-3 or SpiroZin2 with or without pluronic as described above. Nuclear dye was added to the cell culture medium 30 min prior to imaging. After staining, cells were washed with phosphate-free HHBSS buffer. To enhance the fluorescence intensities of the zinc sensor channels, 5 μM pyrithione/20 μM ZnCl_2_ was added to the cells. Z-series fluorescence images were recorded 5–10 min after Zn^2+^ addition on a Nikon Ti-E spinning disc confocal microscope as described in the “Live Cell Imaging” section. Image analysis was performed using ImageJ. Z-series images of each fluorescence channel were flattened using the maximum intensity Z-projection.

Cells with cytosolic distribution of dyes were counted by eye. The total number of cells was calculated with ImageJ. Nucleus channel images were thresholded to identify the nuclear area. Touching objects were separated with a watershed algorithm. The number of nuclei (i.e. cells) was determined with the command “analyze particles.” The quantification of cytosolic distribution of dyes was determined by dividing the number of cells with cytosolic signal by the total number of cells per image.

### Lysosomal pH Measurement

HC11 cells were grown in a 96-well plate in growth media supplemented with 0.5 mg/mL of LysoSensor^TM^ Yellow/Blue Dextran (ThermoFisher Scientific, L22460) overnight. The standard curve was generated by incubating cells in 10 μM monensin and 10 μM nigericin in MES buffer (5 mM NaCl, 115 mM KCl, 1.3 mM MgSO_4_, 25 mM MES), with the pH adjusted to within the range of 4.0–7.0 for 10 min. After the incubation, fluorescence was quantified with a fluorescence microplate reader (BioTek Synergy H1 hybrid) at emission wavelengths of 535 and 440 nm with excitation at 340 nm. To generate a standard curve, the ratio of emission 535 nm/440 nm was plotted against the pH value of MES buffer. The lysosomal pH post Bafilomycin A1 or DMSO treatment was calculated using the standard curve.

### TRPML1 Activation by ML-SA1

To monitor Zn^2+^ release from lysosomes, 8 × 10^5^ HC11 cells were plated in a 35-mm glass bottom dish one day prior to imaging. Prior to imaging, cells were stained with SpiroZin2 and nuclear dye as described above, and then imaged on a Nikon A1R laser scanning confocal microscope as described in the “Live Cell Imaging” section. Z-series images were recorded every 3 min. ML-SA1 (final concentration 20 μM) or DMSO was added to cells for 30 min and then washed off, followed by 12 min treatment with 150 μM TPA. Image analysis was performed with ImageJ. Z-series images of each fluorescence channel per time point were flattened using the maximum intensity Z-projection. Single cell analysis was performed as described in the “Variability of Fluorescence Intensities of Zinc Probes”. Fluorescence after ML-SA1/DMSO treatment was defined as F and fluorescence after TPA treatment was used as F_min_. The lysosomal Zn^2+^ level after ML-SA1 or DMSO treatment is represented as F/F_min_.

### Monitoring Cytosolic Zn^2+^ Using a Genetically-encoded Zn^2+^ FRET Sensor

HC11 cells stably expressing a Zn^2+^ FRET Sensor (ZapCV2) were generated using the PiggyBac™ Transposon Vector System (System Biosciences). Fluorescence images were acquired in the YFP FRET and CFP channels. To perform data analysis, a cytoplasmic region (ROI) and a region without cells (background) were selected and the FRET ratio was calculated by dividing the background corrected YFP FRET intensity by the background corrected CFP FRET intensity.

### Measurement of Lysosomal Zn^2+^ Level During Lactation

HC11 cells were induced to differentiation as described above. To study the change in lysosomal Zn^2+^ over the progression of lactation, cells at different stages were stained with SpiroZin2 (without pluronic) and nuclear dye as described above, and then imaged on a Nikon A1R laser scanning confocal microscope as described in the “Live Cell Imaging” section. Z-series images were recorded in the resting state (F) and 15 min after TPA treatment (F_min_). Image analysis was performed with ImageJ. Z-series images of each fluorescence channel per time point were flattened using the maximum intensity Z-projection. To perform single cell analyses, the same workflow as described in the above section “Variability of Fluorescence Intensities of Zinc Probes” was used: nucleus channel images were analyzed to identify the nucleus area, segment cells, and create ROIs, with each ROI identifying a single cell. Applying the ROIs to the Spirozin2 channel allowed us to quantify the mean intensity of SpiroZin2 at the single-cell level in an entire field of view. Background fluorescence of each channel was calculated by averaging the signal intensities of an area (mean intensities) without cells. The fluorescence intensities of the SpiroZin2 channel of each cell were calculated by subtracting background fluorescence from the raw mean fluorescence intensities. Data plots and statistical analyses (ANOVA with Tukey’s HSD posthoc test) were generated with KaleidaGraph software.

## Electronic supplementary material

Supplementary Information

## References

[1] Kambe T, Tsuji T, Hashimoto A, Itsumura N (2015). The Physiological, Biochemical, and Molecular Roles of Zinc Transporters in Zinc Homeostasis and Metabolism. Physiol. Rev..

[2] Andreini C, Banci L, Bertini I, Rosato A (2006). Counting the zinc-proteins encoded in the human genome. J. Proteome Res..

[3] Krężel A, Maret W (2016). The biological inorganic chemistry of zinc ions. Arch. Biochem. Biophys..

[4] Maret Wolfgang (2017). Zinc in Cellular Regulation: The Nature and Significance of “Zinc Signals”. International Journal of Molecular Sciences.

[5] Carpenter MC, Lo MN, Palmer AE (2016). Techniques for measuring cellular zinc. Arch. Biochem. Biophys..

[6] Anderson CT, Kumar M, Xiong S, Tzounopoulos T (2017). Cell-specific gain modulation by synaptically released zinc in cortical circuits of audition. eLife.

[7] Qiu Mei, Shentu Yang-ping, Zeng Ji, Wang Xiao-chuan, Yan Xiong, Zhou Xin-wen, Jing Xiao-peng, Wang Qun, Man Heng-ye, Wang Jian-zhi, Liu Rong (2017). Zinc mediates the neuronal activity–dependent anti-apoptotic effect. PLOS ONE.

[8] Dittmer PJ, Miranda JG, Gorski JA, Palmer AE (2009). Genetically Encoded Sensors to Elucidate Spatial Distribution of Cellular Zinc. J. Biol. Chem..

[9] Hwang JJ, Lee S-J, Kim T-Y, Cho J-H, Koh J-Y (2008). Zinc and 4-hydroxy-2-nonenal mediate lysosomal membrane permeabilization induced by H2O2 in cultured hippocampal neurons. J. Neurosci. Off. J. Soc. Neurosci..

[10] Eichelsdoerfer JL, Evans JA, Slaugenhaupt SA, Cuajungco MP (2010). Zinc dyshomeostasis is linked with the loss of mucolipidosis IV-associated TRPML1 ion channel. J. Biol. Chem..

[11] Que EL (2015). Quantitative mapping of zinc fluxes in the mammalian egg reveals the origin of fertilization-induced zinc sparks. Nat. Chem..

[12] Taylor KM, Hiscox S, Nicholson RI, Hogstrand C, Kille P (2012). Protein kinase CK2 triggers cytosolic zinc signaling pathways by phosphorylation of zinc channel ZIP7. Sci. Signal..

[13] McCormick N, Velasquez V, Finney L, Vogt S, Kelleher SL (2010). X-Ray Fluorescence Microscopy Reveals Accumulation and Secretion of Discrete Intracellular Zinc Pools in the Lactating Mouse Mammary Gland. PLOS ONE.

[14] Qin Y, Dittmer PJ, Park JG, Jansen KB, Palmer AE (2011). Measuring steady-state and dynamic endoplasmic reticulum and Golgi Zn2+ with genetically encoded sensors. Proc. Natl. Acad. Sci..

[15] Park JG, Qin Y, Galati DF, Palmer AE (2012). New Sensors for Quantitative Measurement of Mitochondrial Zn2+. ACS Chem. Biol..

[16] Miranda Jose G., Weaver Amanda L., Qin Yan, Park J. Genevieve, Stoddard Caitlin I., Lin Michael Z., Palmer Amy E. (2012). New Alternately Colored FRET Sensors for Simultaneous Monitoring of Zn2+ in Multiple Cellular Locations. PLoS ONE.

[17] Hessels AM (2015). eZinCh-2: A Versatile, Genetically Encoded FRET Sensor for Cytosolic and Intraorganelle Zn2+ Imaging. ACS Chem. Biol..

[18] Vinkenborg JL (2009). Genetically encoded FRET sensors to monitor intracellular Zn2+ homeostasis. Nat. Methods.

[19] Chabosseau P (2014). Mitochondrial and ER-Targeted eCALWY Probes Reveal High Levels of Free Zn2+. ACS Chem. Biol..

[20] Zalewski PD, Forbes IJ, Betts WH (1993). Correlation of apoptosis with change in intracellular labile Zn(II) using zinquin [(2-methyl-8-p-toluenesulphonamido-6-quinolyloxy)acetic acid], a new specific fluorescent probe for Zn(II). Biochem. J..

[21] Gee KR, Zhou Z-L, Ton-That D, Sensi SL, Weiss JH (2002). Measuring zinc in living cells.: A new generation of sensitive and selective fluorescent probes. Cell Calcium.

[22] Rivera-Fuentes P, Lippard SJ (2014). SpiroZin1: A Reversible and pH-Insensitive, Reaction-based, Red-fluorescent Probe for Imaging Biological Mobile Zinc. ChemMedChem.

[23] Rivera-Fuentes P (2015). A far-red emitting probe for unambiguous detection of mobile zinc in acidic vesicles and deep tissue. Chem. Sci..

[24] Chung H (2009). Ethambutol-induced toxicity is mediated by zinc and lysosomal membrane permeabilization in cultured retinal cells. Toxicol. Appl. Pharmacol..

[25] Qin Y (2013). Direct Comparison of a Genetically Encoded Sensor and Small Molecule Indicator: Implications for Quantification of Cytosolic Zn2+. ACS Chem. Biol..

[26] Taylor KM (2008). ZIP7-mediated intracellular zinc transport contributes to aberrant growth factor signaling in antihormone-resistant breast cancer Cells. Endocrinology.

[27] Liu M (2013). ZIP8 Regulates Host Defense through Zinc-Mediated Inhibition of NF-κB. Cell Rep..

[28] Suh SW, Gum ET, Hamby AM, Chan PH, Swanson RA (2007). Hypoglycemic neuronal death is triggered by glucose reperfusion and activation of neuronal NADPH oxidase. J. Clin. Invest..

[29] Jayaraman AK, Jayaraman S (2011). Increased level of exogenous zinc induces cytotoxicity and up-regulates the expression of the ZnT-1 zinc transporter gene in pancreatic cancer cells. J. Nutr. Biochem..

[30] Chandler P (2016). Subtype-specific accumulation of intracellular zinc pools is associated with the malignant phenotype in breast cancer. Mol. Cancer.

[31] Wiggins HL (2015). Disulfiram-induced cytotoxicity and endo-lysosomal sequestration of zinc in breast cancer cells. Biochem. Pharmacol..

[32] Roh HC, Collier S, Guthrie J, Robertson JD, Kornfeld K (2012). Lysosome-related organelles in intestinal cells are a zinc storage site in C. elegans. Cell Metab..

[33] Taniguchi M (2013). Essential Role of the Zinc Transporter ZIP9/SLC39A9 in Regulating the Activations of Akt and Erk in B-Cell Receptor Signaling Pathway in DT40 Cells. PLOS ONE.

[34] Rohrer J, Schweizer A, Russell D, Kornfeld S (1996). The targeting of Lamp1 to lysosomes is dependent on the spacing of its cytoplasmic tail tyrosine sorting motif relative to the membrane. J. Cell Biol..

[35] Wang C-C (2004). A role of VAMP8/endobrevin in regulated exocytosis of pancreatic acinar cells. Dev. Cell.

[36] Lippert U, Ferrari DM, Jahn R (2007). Endobrevin/VAMP8 mediates exocytotic release of hexosaminidase from rat basophilic leukaemia cells. FEBS Lett..

[37] Loo LS (2009). A role for endobrevin/VAMP8 in CTL lytic granule exocytosis. Eur. J. Immunol..

[38] Polgár J, Chung S-H, Reed GL (2002). Vesicle-associated membrane protein 3 (VAMP-3) and VAMP-8 are present in human platelets and are required for granule secretion. Blood.

[39] Wang C-C (2010). A role for VAMP8/endobrevin in surface deployment of the water channel aquaporin 2. Mol. Cell. Biol..

[40] Bolte S, Cordelières FP (2006). A guided tour into subcellular colocalization analysis in light microscopy. J. Microsc..

[41] Fiedler BL (2017). Droplet Microfluidic Flow Cytometer For Sorting On Transient Cellular Responses Of Genetically-Encoded Sensors. Anal. Chem..

[42] Ehrlich M (2004). Endocytosis by Random Initiation and Stabilization of Clathrin-Coated Pits. Cell.

[43] Kukic I, Lee JK, Coblentz J, Kelleher SL, Kiselyov K (2013). Zinc-dependent lysosomal enlargement in TRPML1-deficient cells involves MTF-1 transcription factor and ZnT4 (Slc30a4) transporter. Biochem. J..

[44] Kukic I, Kelleher SL, Kiselyov K (2014). Zn2+ efflux through lysosomal exocytosis prevents Zn2+ -induced toxicity. J. Cell Sci..

[45] Shen D (2012). Lipid storage disorders block lysosomal trafficking by inhibiting a TRP channel and lysosomal calcium release. Nat. Commun..

[46] Gómez NM (2018). Robust lysosomal calcium signaling through channel TRPML1 is impaired by lysosomal lipid accumulation. FASEB J..

[47] Zou J (2015). Reactivation of Lysosomal Ca2+ Efflux Rescues Abnormal Lysosomal Storage in FIG4-Deficient Cells. J. Neurosci..

[48] Yoshimori T, Yamamoto A, Moriyama Y, Futai M, Tashiro Y (1991). Bafilomycin A1, a specific inhibitor of vacuolar-type H(+)-ATPase, inhibits acidification and protein degradation in lysosomes of cultured cells. J. Biol. Chem..

[49] Montanini B, Blaudez D, Jeandroz S, Sanders D, Chalot M (2007). Phylogenetic and functional analysis of the Cation Diffusion Facilitator (CDF) family: improved signature and prediction of substrate specificity. BMC Genomics.

[50] Lu M, Chai J, Fu D (2009). Structural basis for autoregulation of the zinc transporter YiiP. Nat. Struct. Mol. Biol..

[51] Grass G (2005). FieF (YiiP) from Escherichia coli mediates decreased cellular accumulation of iron and relieves iron stress. Arch. Microbiol..

[52] Shusterman E (2014). ZnT-1 extrudes zinc from mammalian cells functioning as a Zn2+/H+ exchanger. Metallomics.

[53] Ohana E (2009). Identification of the Zn2+ Binding Site and Mode of Operation of a Mammalian Zn2+ Transporter. J. Biol. Chem..

[54] Palmiter RD, Cole TB, Findley SD (1996). ZnT-2, a mammalian protein that confers resistance to zinc by facilitating vesicular sequestration. EMBO J..

[55] Falcón-Pérez JM, Dell’Angelica EC (2007). Zinc transporter 2 (SLC30A2) can suppress the vesicular zinc defect of adaptor protein 3-depleted fibroblasts by promoting zinc accumulation in lysosomes. Exp. Cell Res..

[56] Ball RK, Friis RR, Schoenenberger CA, Doppler W, Groner B (1988). Prolactin regulation of beta-casein gene expression and of a cytosolic 120-kd protein in a cloned mouse mammary epithelial cell line. EMBO J..

[57] Cella N, Groner B, Hynes NE (1998). Characterization of Stat5a and Stat5b homodimers and heterodimers and their association with the glucocortiocoid receptor in mammary cells. Mol. Cell. Biol..

[58] Carter KP, Young AM, Palmer AE (2014). Fluorescent Sensors for Measuring Metal Ions in Living Systems. Chem. Rev..

[59] Lee S-J, Koh J-Y (2010). Roles of zinc and metallothionein-3 in oxidative stress-induced lysosomal dysfunction, cell death, and autophagy in neurons and astrocytes. Mol. Brain.

[60] Cho KS, Yoon YH, Choi JA, Lee S-J, Koh J-Y (2012). Induction of autophagy and cell death by tamoxifen in cultured retinal pigment epithelial and photoreceptor cells. Invest. Ophthalmol. Vis. Sci..

[61] Lee S-J, Cho KS, Koh J-Y (2009). Oxidative injury triggers autophagy in astrocytes: the role of endogenous zinc. Glia.

[62] Hwang JJ (2010). Zinc(II) ion mediates tamoxifen-induced autophagy and cell death in MCF-7 breast cancer cell line. Biometals.

[63] Rivera OC, Hennigar SR, Kelleher SL (2016). Loss of ZnT2-mediated Zinc Transport Leads to Cytoplasmic Zinc Accumulation and Impairs Mammary Gland Involution. FASEB J..

[64] Hennigar SR, Seo YA, Sharma S, Soybel DI, Kelleher SL (2015). ZnT2 is a critical mediator of lysosomal-mediated cell death during early mammary gland involution. Sci. Rep..

[65] Hennigar SR, Lanz MC, Kelleher SL (2013). TNFα redistributes ZnT2 to accumulate zinc in lysosomes and activate autophagic cell death in mammary epithelial cells. FASEB J..

