# Supplementary material for: Superiority of SpiroZin2 Versus FluoZin-3 for monitoring vesicular Zn2+ allows tracking of lysosomal Zn2+ pools
Source: Sci Rep. 2018 Oct 9;8:15034. doi: 10.1038/s41598-018-33102-w (PMC6177427; doi:10.1038/s41598-018-33102-w)
Supplement: Supplementary file 1 — Supplementary Information [file 41598_2018_33102_MOESM1_ESM.pdf]

Supplementary information for:

Superiority of SpiroZin2 Versus FluoZin-3 for monitoring vesicular Zn^{2+} allows tracking of lysosomal Zn^{2+} pools

Yu Han[1], Jacob M. Goldberg[2], Stephen J. Lippard[2], Amy E. Palmer[1*]

1 Department of Chemistry and Biochemistry and BioFrontiers Institute, University of Colorado, Boulder CO 80303
2 Department of Chemistry, Massachusetts Institute of Technology, Cambridge MA 02139-4307

Supplementary information contains 1 supplementary figure.

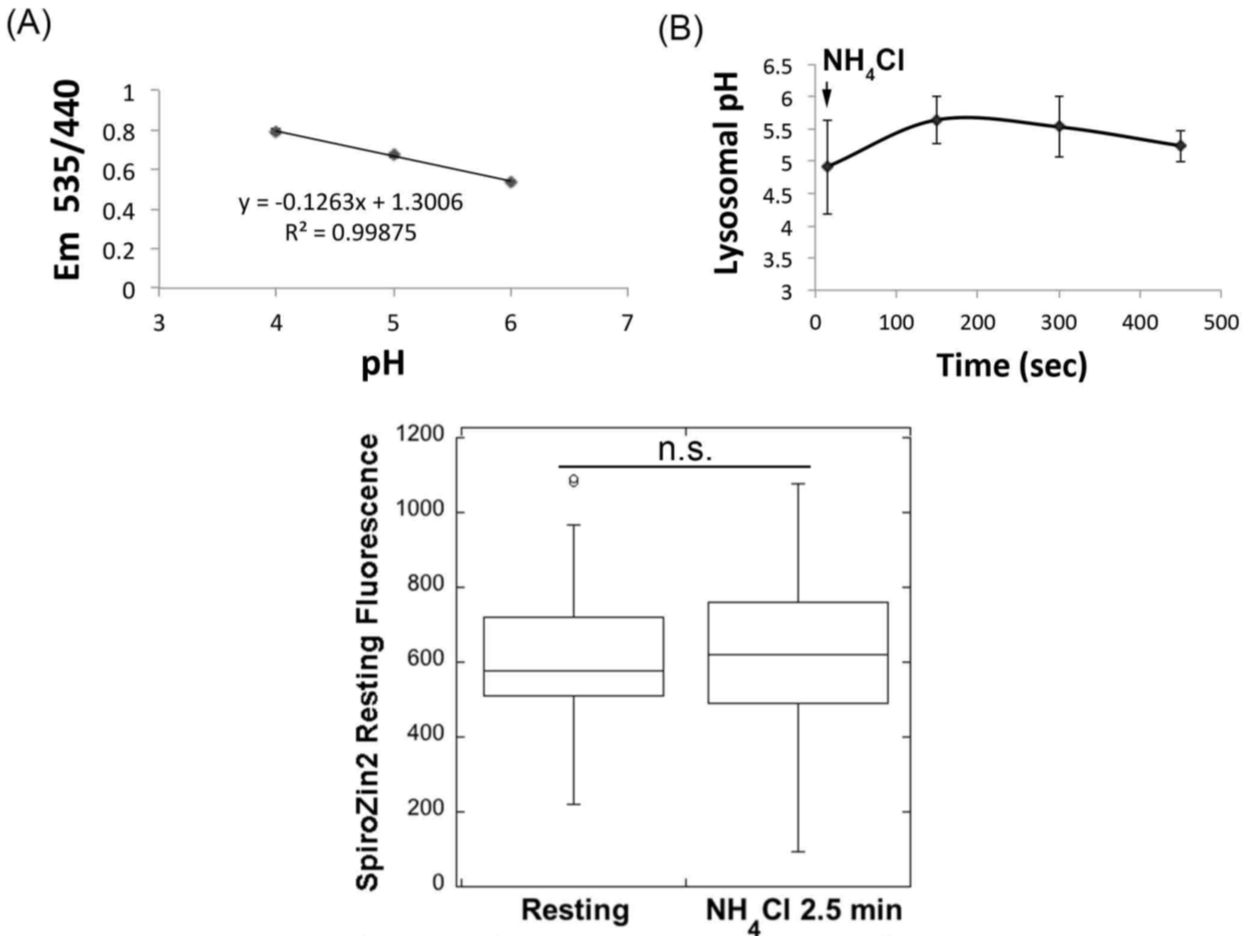

Mean ± SD	610.8 ± 157.0	620.1 ± 189.2

Supplementary Figure 1. SpiroZin2 is insensitive to rapid pH increase in HC11 cells. Lysosomal pH was measured as previously described[1]. Briefly, HC11 cells were grown in a 96-well plate in growth media supplemented with 0.5 mg / mL of LysoSensor^{TM} Yellow / Blue Dextran (ThermoFisher Scientific, L22460) overnight. The standard curve (A) was generated by incubating cells in 10 uM monensin and 10 μM nigericin in MES buffer (5 mM NaCl, 115 mM KCl, 1.3 mM $MgSO_4$, 25 mM MES), with the pH adjusted to within the range of 4.0–7.0 for 10 min. After the incubation, fluorescence was quantified with a fluorescence microplate reader (BioTek Synergy H1 hybrid) at an emission wavelength of 535 and 440 nm with excitation at 340 nm. The ratio of emission 535nm / 440 nm was plotted against the pH value of MES buffer. To test the in situ pH sensitivity of SpiroZin2, first, the effect of NH_4Cl treatment on lysosomal pH was investigated. Briefly, HC11 cells were loaded with 0.5 mg / mL of the pH sensor overnight. Cells were washed with phosphate-free HHBSS buffer and then 10 mM of NH_4Cl was added to cell media. The ratio of emission 535 / 440 was recorded at 15 sec, 2.5 min, 5 min and 7.5 min post NH_4Cl treatment and then was converted to lysosomal pH using the standard curve (B). Figure 1 (B) showed that lysosomal pH peaked at 2.5 min post treatment (pH = 5.64). To test the pH sensitivity of SpiroZin2, HC11 cells were stained with SpiroZin2 and then treated with 10

mM of NH_4Cl. Resting fluorescence of SpiroZin2 was recorded prior to (resting) and 2.5 min post treatment (NH_4Cl 2.5 min), as shown in Figure 1 (C). Statistical analysis showed that the pH increase had no effect on SpiroZin2 signal (n.s., not significant, paired student's t-test, $N = 175$ cells).

Reference

1. Yanagawa, M. *et al.* Cathepsin E Deficiency Induces a Novel Form of Lysosomal Storage Disorder Showing the Accumulation of Lysosomal Membrane Sialoglycoproteins and the Elevation of Lysosomal pH in Macrophages. *J. Biol. Chem.* **282,** 1851–1862 (2007).
